# A Slow-Growing Hand Mass

**Published:** 2018-03-08

**Authors:** Erica Y. Xue, Ronald Bogdasarian, Ramazi Datiashvili

**Affiliations:** Division of Plastic and Reconstructive Surgery, Department of Surgery, Rutgers New Jersey Medical School, Newark

**Keywords:** hand mass, hand tumor, repetitive trauma, hand tumor epidemiology, hand mass diagnosis and management

## DESCRIPTION

A 44-year-old right-handed male construction worker presented with a slow-growing, firm, soft-tissue mass at the palm between the middle and ring finger metacarpals heads. The mass was causing pain, morning stiffness, and interference with work.

## QUESTIONS

1. What is the differential diagnosis of a slow-growing hand mass?
2. What is the role of radiographic imaging in diagnosing hand masses?
3. What is the role of occupation and repetitive trauma in the development of this lesion?
4. What anatomic structures should be avoided intraoperatively during excision of the mass?

## DISCUSSION

Benign lesions can arise from all tissue types in the hand—skin, fat, muscle, nerve, vessel, and more. The most common benign soft-tissue tumors of the hand are ganglion cysts and giant cell tumors of the tendon sheath. Ganglion cysts are more prevalent in women, and 70% occur between the second and fourth decades of life. Giant cell tumors of the tendon sheath are the second most common tumors seen in the hand. These tumors are slow-growing and may be asymptomatic unless the lesion occurs in proximity to the digital nerves, resulting in a sensory deficit.^1^ Glomus tumors and peripheral nerve tumors are classic volar hand tumors to consider in the differential diagnosis. When clinically assessing a hand mass, time frame, location, size, mobility, pain, tenderness, and previous trauma are important etiologic clues.

For most patients with simple subcutaneous hand masses, imaging will not alter management, as excision and pathology are the criterion standards for diagnosis. However, imaging provides clues for diagnosis and may guide excision. Imaging may demonstrate tissue type, mass encapsulation, invasion, and proximity to surrounding anatomic structures. There are a variety of imaging modalities available for the assessment of hand masses. In the basic hand radiograph, 3 views are obtained: anterior-posterior, oblique, and lateral. Standard radiography determines whether or not the lesion developed from the osseous elements as well as the presence of subcutaneous calcifications.^2^ Ultrasonography is particularly useful for analyzing cystic nature, vascularity, and possible vein thrombosis. Computed tomography and magnetic resonance imaging are second-line modalities for further soft-tissue and tumor evaluation.^2^ Our patient's radiographs demonstrated normal soft tissues, no radiopaque foreign body, no erosive changes, or ectopic calcification. Ultrasound scan described a hypoechoic mass between the third and fourth metacarpal joints, adjacent to the flexor tendon.

Based on clinical presentation, the mass was thought to be a giant cell tumor of the tendon sheath. However, the final pathology report described it as densely fibrotic tissue with chronic inflammation. The lesion measured 4.3 × 3 × 1 cm. The gross appearance of the mass was flesh-colored, smooth, and homogeneous. The inflammation and fibrosis found on pathology may be explained by the pathophysiology of work-related musculoskeletal disorders of the hand and wrist. Basic science studies in animal and human models suggest that inflammatory changes are more prevalent than degenerative changes in overuse injuries; these inflammatory and proliferative changes appear early before overt signs of tissue injury or tendinopathy.^3^ Furthermore, in models of chronic repetitive motion, inflammation eventually subsides and is followed by a fibrotic response, which likely led to our patient's symptomatic hand tumor.^4^

Definitive treatment of most hand masses is excision. The common and proper neurovascular structures were identified and protected during the dissection of the mass. In the hand, arteries run volar to nerves in the palm and dorsal to the nerves in the digits. The superficial and deep palmar arterial arches give off the common digital arteries in the central palm and proper digital arteries at the metacarpophalangeal joint level. The mass in question was located at the transition between these structures.

The hand is a highly utilized appendage that can give rise to various benign masses. Based on clinical examination, hand masses have a broad differential diagnosis that may ultimately have an uncommon pathology, such as fibrotic tissue. A number of diagnostic imaging modalities can be utilized in assessing hand masses. Ultimately, surgical excision is indicated for treatment and diagnosis. During excision, impeccable attention to anatomy is essential to preserve normal structure and function.

## Figures and Tables

**Figure 1:**
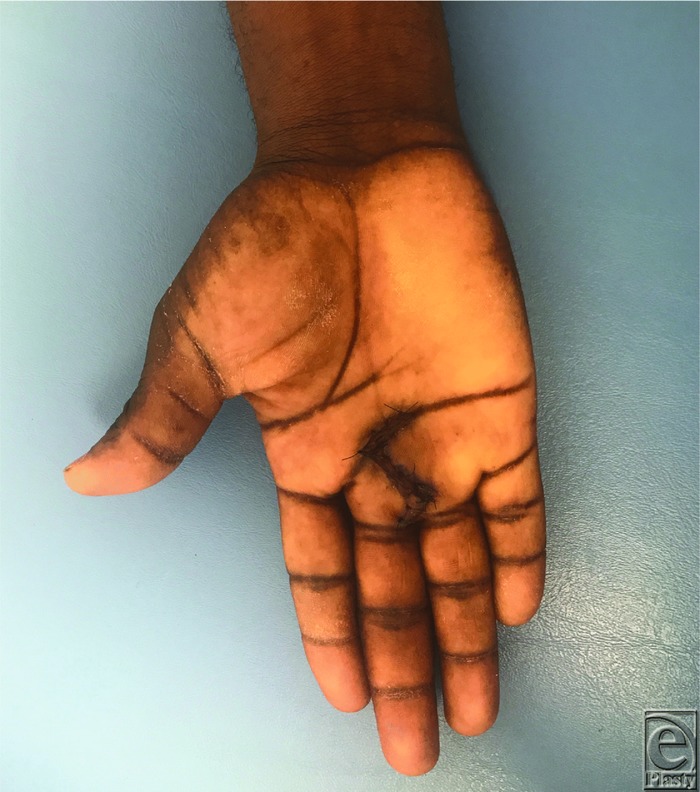
Two weeks postoperatively.
